# Treatment outcomes for people with hepatitis C referred to tertiary care in Victoria, 2021–22: a retrospective observational study

**DOI:** 10.5694/mja2.70017

**Published:** 2025-08-11

**Authors:** Elly Layton, Nicole Matthews, Brendan Quinn, Nasra Higgins, Gabrielle Lindeman, Mielle Abbott, Jennifer MacLachlan, Elizabeth Birbilis, Margaret E Hellard, Joseph Doyle, Benjamin C Cowie, Mark Stoové

**Affiliations:** ^1^ Burnet Institute Melbourne VIC; ^2^ Department of Health Victoria Melbourne VIC; ^3^ WHO Collaborating Centre for Viral Hepatitis the Peter Doherty Institute for Infection and Immunity Melbourne VIC; ^4^ The University of Melbourne Melbourne VIC; ^5^ Victorian Infectious Diseases Service the Royal Melbourne Hospital Melbourne VIC; ^6^ Monash University Melbourne VIC; ^7^ Australian Research Centre in Sex, Health and Society La Trobe University Melbourne VIC

**Keywords:** Hepatitis C, Public health, Treatment outcome, General practice

In Australia, an estimated 68 890 people were living with chronic hepatitis C virus (HCV) infections at the end of 2023.^1^ An overwhelming majority of incident HCV infections in Australia are in people who inject drugs.^2^ In alignment with World Health Organization targets, the Australian government has committed to eliminating HCV as a public health threat by 2030.^3^ In 2016, Australia became one of the first countries in which direct‐acting antiviral (DAA) medications were broadly available for the treatment of HCV infections, including through primary care clinicians, and HCV elimination strategies emphasise the importance of general practitioner prescribing.^3^ However, current treatment rates are slowing progress to elimination;^4^ in 2023, treatment was initiated for only 5499 people living with HCV infection.^1^ The declining initiation of treatment by specialist medical practitioners has not been offset by initiations by general practitioners and nurse practitioners.^1^

In the Coordinated Hepatitis response to Enhance the Cascade of Care (CHECCS) project, public health officers followed up clinicians to support their care for people they diagnosed with HCV infections and notified to the Victorian Department of Health during 1 September 2021 – 31 March 2022.^5^ Despite HCV‐related strategic priorities that encourage treatment in primary care, diagnosing clinicians reported referring 50 of the 117 people positive for HCV RNA during the study period (43%) to specialist care (ie, not general practitioners).^5^ This finding was consistent with the national pattern of HCV treatment prescribing.^6^

Little is known about the clinical outcomes of DAA treatment not initiated by general practitioners.^7^ We therefore assessed treatment uptake by the 50 HCV RNA‐positive people referred to tertiary care clinics during the CHECCS project. During 26 July – 26 September 2024, we contacted the tertiary care clinics to which people had been referred to ascertain their appointment attendance, treatment, and whether sustained virological response (SVR) had been achieved. We also describe the demographic characteristics and HCV infection risk factors for people who were treated after referral. Our study was approved by the Alfred Hospital Ethics Committee (project 61/24).

Of the 50 people referred to tertiary specialist clinics, 44 had been diagnosed with HCV infections in general practices and six in hospitals. We could follow up tertiary care for 45 people; their median age was 53 years (interquartile range [IQR], 39–64 years), and 29 were men. Scheduled appointments were recorded for 37 people; of the eight people without scheduled appointments, three had been referred but were eventually treated by the diagnosing clinicians, one person with an inpatient referral was treated while still in hospital, referrals were not recorded for two people, and two were on clinic waiting lists (about 16 and 21 months after being referred). Thirty‐two of 37 people with appointments attended the appointments, and 28 commenced DAA treatment. At the time we contacted the clinics, 26 people had completed treatment, one was still receiving treatment, and one had been lost to follow‐up. Of the four people who attended appointments but were not offered treatment, the infection had spontaneously cleared in two, one was not eligible for Medicare cover, and one required further investigations. Of the 26 people who completed treatment, evidence of SVR was reported for 24; one person required further treatment, and one had not been assessed for SVR (Box 1). Twenty‐two people who attended clinics had been investigated for cirrhosis; six (including five men) were diagnosed with cirrhosis (median age, 66 years; IQR, 61–67 years).

Box 1 **Cascade of tertiary care for fifty hepatitis C virus (HCV) RNA‐positive people referred to tertiary care in Victoria, 1 September 2021 – 31 March 2022**

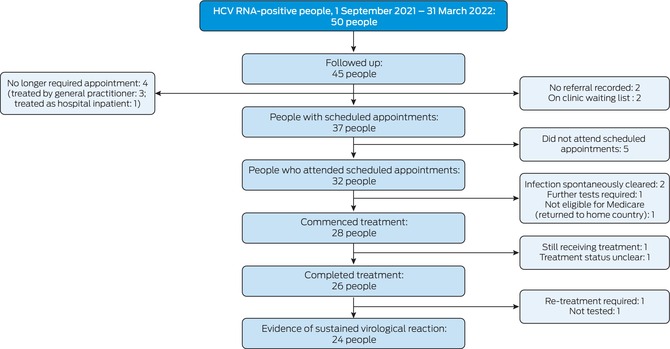

Of 37 people with HCV infections referred to tertiary clinics, 24 (65%) commenced treatment and achieved SVR. The proportion of men who commenced treatment was slightly larger than for women; it was larger for people aged 40 years or older than for those under 40 years of age, and smaller among people who reported injecting drugs during the preceding two years (Box 2).

The age profile of the people referred to tertiary care in our study, and the larger proportion of people aged 40 years or older who commenced treatment, could be linked with their greater risk of advanced liver disease and cirrhosis (older people may have lived longer with chronic HCV infection); medical specialist care is recommended for such people.^8^ But most people in our study could have been treated in primary care; only a few were diagnosed with cirrhosis.

All fifteen treatment‐eligible people without histories of injecting drug use commenced treatment, as did seven of eight who reported injecting drug use but not during the preceding two years, but only one of five people who reported more recent injecting drug use (Box 2). Reasons for low treatment uptake in this third group included not attending appointments or being placed on clinic waiting lists (data not shown). An Australian randomised controlled trial found that DAA uptake by people who inject drugs was greater in primary care (43 of 57, 75%) than in tertiary care (18 of 53, 34%).^9^ People who inject drugs may experience stigmatisation by hospital staff.^10^ Our findings indicate that, for some people, referral to tertiary care for HCV treatment can be successful, but the choice of referral pathway should take the individual into account. Greater awareness among diagnosing clinicians of non‐tertiary HCV care referral pathways, including care integrated into primary care services specialising in care for people who use drugs, would support DAA uptake and HCV elimination strategies.^11^

Box 2 **Demographic characteristics of fifty hepatitis C virus (HCV) RNA‐positive people referred to tertiary care in Victoria, 1 September 2021 – 31 March 2022, by treatment eligibility and uptake**

**Table:** 

Characteristic	People who could be followed up	Treatment‐eligible*	Treatment‐eligible, commenced treatment^†^
Total	45	41	32
Sex^‡^			
Male	29	26	21
Female	16	15	11
Country of birth			
Australia	16	14	11
Overseas	14	13	12
Not reported	15	14	9
Age (years)			
Under 40	12	10	5
40 or older	33	31	27
Injecting drug use			
Yes, not in preceding two years	9	8	7
Yes, in preceding two years	6	5	1
No	16	15	15
Not reported	14	13	9

* Excludes people in whom the HCV infection had spontaneously cleared, who were not eligible for Medicare, or for whom further investigations were in progress at the time of follow‐up.

† Includes people treated in tertiary care or elsewhere.

‡ Based on electronic laboratory records; for surveillance purpose, this is interpreted as biological sex at birth or current sex attributes as recorded in clinic patient management systems.

Our study was limited by its small sample size and incomplete follow‐up of all eligible people. However, our findings regarding the cascade of care and the treatment outcomes for people with HCV infections referred to tertiary hospitals indicate important health system strengths and weaknesses. Our findings could inform clinical practice and HCV elimination strategies in Australia.

## Open access

Open access publishing facilitated by Monash University, as part of the Wiley – Monash University agreement via the Council of Australian University Librarians.

## Competing interests

Mark Stoové has received investigator‐initiated research funding from Gilead Sciences and AbbVie and consultant fees from Gilead Sciences for activities unrelated to this work. Margaret Hellard receives funding from Gilead Science and AbbVie for investigator‐initiated research related to hepatitis C.

## Data sharing

Line‐listed data cannot be shared, in line with ethics approvals.
